# Embryo sac formation and early embryo development in *Agave tequilana* (Asparagaceae)

**DOI:** 10.1186/2193-1801-3-575

**Published:** 2014-10-01

**Authors:** Alejandra G González-Gutiérrez, Antonia Gutiérrez-Mora, Benjamín Rodríguez-Garay

**Affiliations:** Unidad de Biotecnología Vegetal, Centro de Investigación y Asistencia en Tecnología y Diseño del Estado de Jalisco, A.C. (CIATEJ), Av. Normalistas No. 800, Col. Colinas de la Normal, Guadalajara, Jalisco 44270 México

**Keywords:** Agavaceae, Chalazal haustorium, Helobial endosperm, Hypostase, Megagametogenesis, Megasporogenesis, Polygonum-type

## Abstract

*Agave tequilana* is an angiosperm species that belongs to the family Asparagaceae (formerly Agavaceae). Even though there is information regarding to some aspects related to the megagametogenesis of *A. tequilana*, this is the first report describing the complete process of megasporogenesis, megagametogenesis, the early embryo and endosperm development process in detail. The objective of this work was to study and characterize all the above processes and the distinctive morphological changes of the micropylar and chalazal extremes after fertilization in this species. The agave plant material for the present study was collected from commercial plantations in the state of Jalisco, Mexico. Ovules and immature seeds, previously fixed in FAA and kept in ethanol 70%, were stained based on a tissue clarification technique by using a Mayer’s-Hematoxylin solution. The tissue clarification technique was successfully used for the characterization of the megasporogenesis, megagametogenesis, mature embryo sac formation, the early embryo and endosperm development processes by studying intact cells. The embryo sac of *A. tequilana* was confirmed to be of the monosporic Polygonum-type and an helobial endosperm formation. Also, the time-lapse of the developmental processes studied was recorded.

**Electronic supplementary material:**

The online version of this article (doi:10.1186/2193-1801-3-575) contains supplementary material, which is available to authorized users.

## Background

One of the main characteristics of Angiosperms is that they possess seeds enclosed inside a fruit derived from the ovary of flowers (Li and Ma 2002). Another outstanding characteristic of angiosperms is that they present alternation of generations in their life cycle (as in many other plants), that is divided in two phases: one dominant diploid phase, which is called sporophytic, and one haploid phase known as gametophytic (Haig 1990; Rodríguez-Garay et al. 2000; Fan et al. 2008; Ma and Sundaresan 2010). The main function of the gametophyte phase is the production of haploid gametes whether they are male or female (Reiser and Fischer 1993; Yadegari and Drews 2004; Fan et al. 2008).

The female gametophyte, also named megagametophyte or embryo sac, is developed within the carpel, which consists of three elements: the stigma, the style and the ovary, which can contain one or several ovules (Gutiérrez-Mora et al. 2012). In each ovule meiosis of the megaspore mother cell produces four haploid cells called megaspores. In the monosporic pattern, three of these megaspores degenerate while the closest cell to the chalazal region remains viable and gives rise to a single functional megaspore.

During the megagametogenesis process, the functional megaspore passes through one or more mitotic divisions without cytokinesis forming a multinucleate coenocyte. Latter, cell walls are formed around the nuclei resulting in a mature embryo sac (Rabiger and Drews 2013). The embryo sacs may present a diversity of developmental pathways, however, the most common is the monosporic Polygonum-type, in which the functional megaspore passes through three mitotic divisions producing a seven celled embryo sac (Chasan and Walbot 1993; Li and Ma 2002; Maheshwari 1937) consisting of three antipodal cells, one central cell formed by two polar nuclei, two synergid cells, and the egg cell (Dresselhaus 2006; Kägi and Groß-Hardt 2007; Yang et al. 2010).

Some studies have characterized the female gametophyte of different species belonging to the Asparagaceae family formerly Agavaceae (APG III 2009), where the majority of them have been described as Monosporic Polygonum-type. Among these species, *Yucca rupicola* (Watkins 1937); *Y. aloifolia* (Wolf 1940); *Y. filamentosa* (Reed 1903); *Agave lechuguilla* (Grove 1941), *A. virginica* (Regen 1941), *Hesperocallis undulata*, *Leucocrinum montanum* (Cave 1948) and *Comospermum yedoense* (Rudall 1999) are found. However, Piven et al. (2001) reported the embryo sac development of *Agave fourcroydes* and *A. angustifolia* as bisporic Allium-type.

Only one study could be found that was centered on the *Agave tequilana* female gametophyte development where Escobar-Guzmán et al. (2008) reported that the megagametophyte is originated from the megaspore that is located closest to the chalazal region, forming an embryo sac of seven cells (Polygonum-type). However, these authors did not report or describe the whole mitotic division process that gives rise to this sac with seven cells, nor the early embryogenesis. Even though there is information regarding to some aspects related to the megagametogenesis of *A. tequilana*, there is no published information describing the embryo and endosperm development in this species and the distinctive morphological changes of the embryo sac after fertilization.

The objective of this work was to study and characterize the process of megasporogenesis, megagametogenesis, the mature embryo sac formation and the early embryo development in *Agave tequilana* Weber which is the raw material for the production of Tequila in Mexico, in order to get basic knowledge useful for plant systematics and evolution studies and plant breeding programs, which may include *in vitro* fertilization and the production of haploid plants among others.

## Results

**Figure 1:**
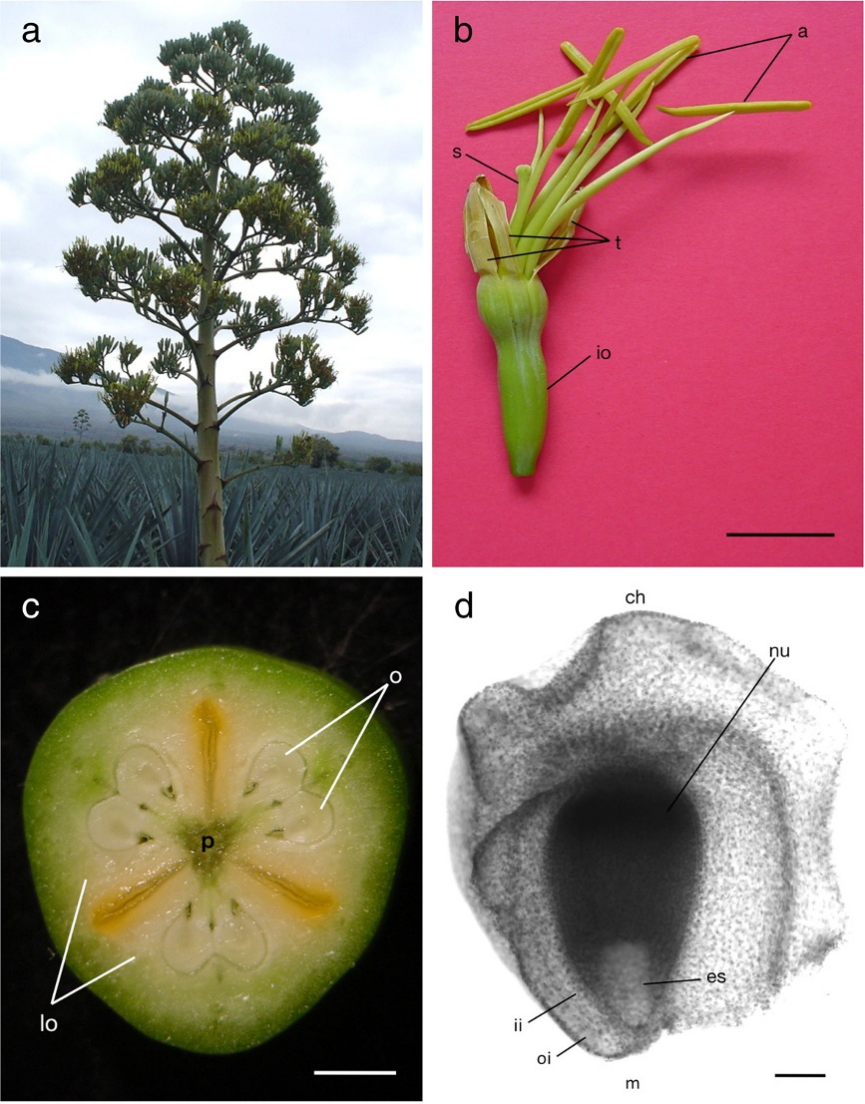
**Plant material of** ***Agave tequilana*** **Weber for the present study. a)** Commercial plantation in the state of Jalisco, México. **b)** Hermafrodite agave flower. Bar = 1 cm. **c)** Agave ovary components. Bar = 2 mm. **d)** Agave immature ovule. Bar = 50 μm. a = anthers, t = tepals, io = inferous ovary, s = style, o = ovules, lo = locules, p = placentae, ch = chalaza, m = micropyle, ii = interior integument, oi = outer integument, n = nucellar tissue, es = embryo sac.

A total of 5,000 ovules were taken from floral buds at diverse stages, receptive flowers and immature fruits were analyzed. Different developmental stages of the collected ovules were studied from megasporogenesis to the first division of the embryo. The plant material used in this study consisted of inflorescences collected from May to June in the years 2010–2013 (Figure 1). It was difficult to know the specific timeframe of each developmental stage of the embryo sac, however, it could be observed that it takes about 15 days for the floral buds since their appearance to reach the maturity of the embryo sac.

### Megasporogenesis

**Figure 2:**
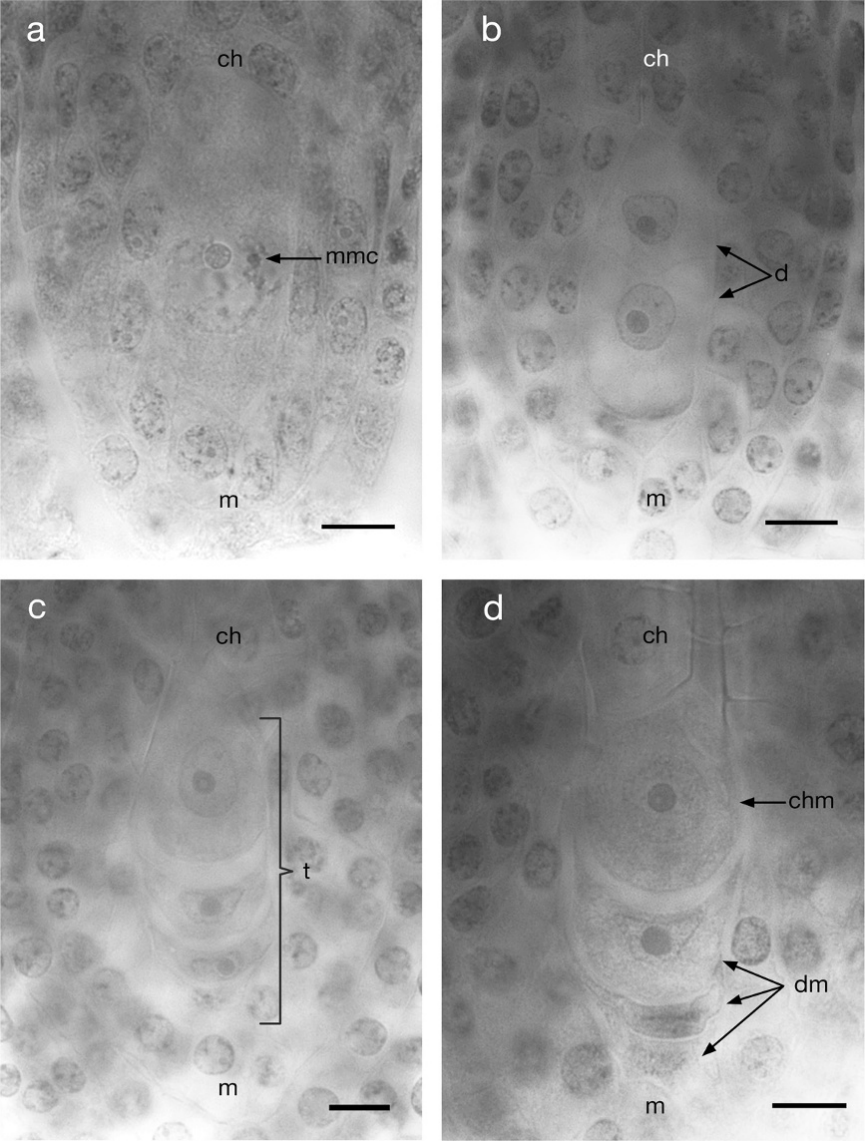
**Megasporogenesis of** ***Agave tequilana*** **Weber. a)** Diploid megaspore mother cell. **b)** Diad after the first meiotic division formed by two haploid cells. **c)** Tetrad after the second meiotic division composed by four haploid cells. **d)** Three degenerating megaspores and one intact functional megaspore located at the chalazal pole of the future embryo sac. ch = chalaza, m = micropyle, mmc = megaspore mother cell, d = dyad, t = tetrad, chm = chalazal megaspore, dm = degenerating megaspores. Bars = 10 μm.

The process of megasporogenesis was observed using ovules in different developmental stages. The first observed stages showed a megaspore mother cell (MMC) differentiation from the nucellar tissue (Figure 2a). This MMC goes through a meiotic cell division that first results in a dyad and finally in a linear tetrad of haploid cells with a chalaza-micropylar orientation (Figure 2b, c), the average size of the tetrad is about 58 μm long and 21 μm wide (for the rest of measurements mentioned on this paper see Additional file 1: Table S1).Once the meiotic tetrad is formed, the three megaspores closest to the micropylar pole degenerate (Figure 2d) and their remains could be observed as highly stained spots which were frequently observed in the two-nucleated embryo sacs. Only the megaspore cell closest to the chalazal pole remains viable, becoming the functional megaspore (FM), and its size is bigger than the rest of the cells in the tetrad with dimensions of about 33 μm long and 23 μm wide.

### Megagametogenesis

**Figure 3:**
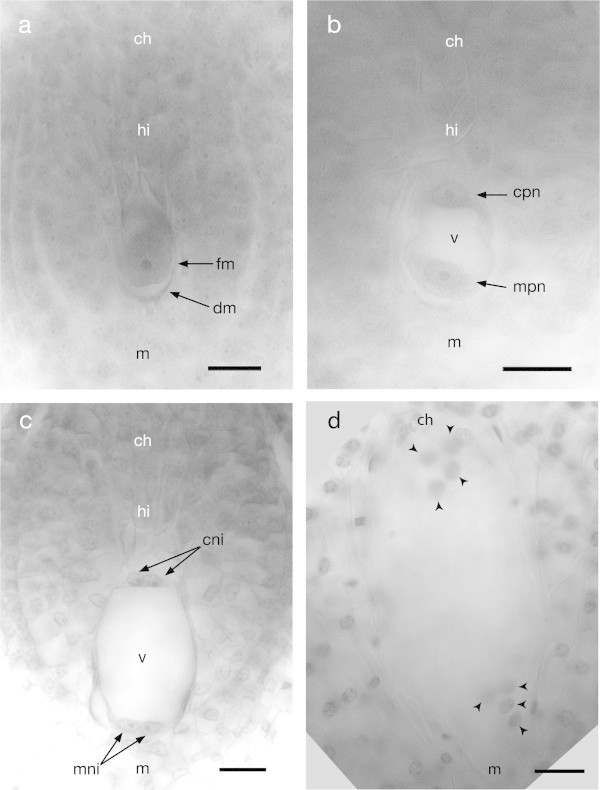
**Megagametogenesis of** ***Agave tequilana*** **Weber. a)** Functional megaspore with the rest of the degenerated megaspores. Bar = 20 μm. **b)** Two nuclei resulting after a first mitotic division of the functional megaspore. Bar = 20 μm. **c)** Four-nuclei state after a second mitotic division. Bar = 20 μm. **d)** Eight-nuclei state forming a multinucleate coenocyte after the third and last mitotic division. Bar = 20 μm. ch = chalaza, hi = hypostase, m = micropyle, fm = functional megaspore, dm = degenerating megaspores, cpn = chalazal polar nucleus, mpn = micropylar polar nucleus, cni = chalazal nuclei, mni = micropylar nuclei. Arrowheads = eight final nuclei.

The process of megagametogenesis starts with the increase in size of the FM, which is about 43 μm long and 25 μm wide. At this stage, the hypostase starts to be visible just above the FM in the nucellar tissue; this structure was detected as a well defined and intensely stained group of cells, which have a poor cytoplasmic content and thickened cell walls (Figure 3a) (also see Additional file 2: Figure S1).The first mitotic division of the functional megaspore produces two nuclei, one located at the chalazal pole and the other one at the micropylar pole, both separated by a large vacuole in the center of the embryo sac without cytokinesis. At this stage, the mean size of the embryo sac is about 50 μm long and 35 μm wide (Figure 3b). Later, these two nuclei divided again forming four nuclei, two located at the chalazal end and the other two at the micropylar end of the embryo sac (Figure 3c). At the same time, the embryo sac continues expanding its size to about 60 μm long and 42 μm wide, and in both stages of embryo sac development its morphology is ovoid. A third and last mitotic division gives place to an eight-nucleated embryo sac (Figure 3d). These three mitotic divisions occur in a synchronized manner at both extremes of the embryo sac.

### The mature embryo sac

**Figure 4:**
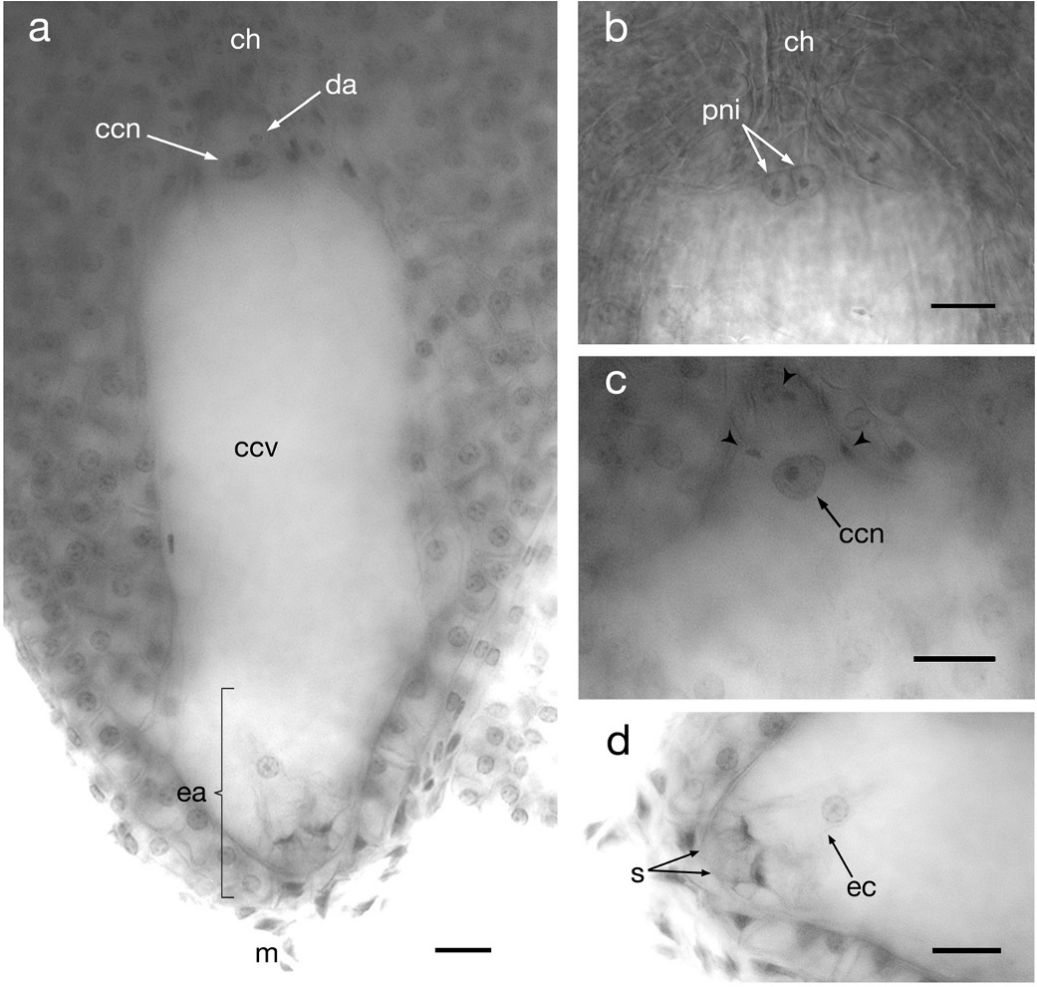
**Mature embryo sac or megagametophyte of** ***Agave tequilana*** **Weber. a)** Whole mature embryo sac showing the contents of both the chalazal and the micropylar poles. **b)** Polar nuclei at the chalazal end before karyogamy. **c)** Central cell nucleus after karyogamy took place. **d)** Egg apparatus composed by two synergid cells and the egg cell. ch = chalaza, ea = egg apparatus, m = micropyle, da = degenerating antipodal cell, ccn = central cell nucleus, ccv = central cell vacuole, pni = polar nuclei, s = synergid cells, ec = egg cell. Arrowheads = degenerating antipodal cells. Bars = 20 μm.

The study of mature embryo sacs was carried out in ovules from flowers with mature and receptive stigma. At the mature stage, the size of the embryo sacs is about 247 μm long and 106 μm wide and they are wider at the chalazal pole than at the micropylar end, having a bulbous form with a small and narrow haustorial tube at the chalazal end which connects to the hypostase (Figure 4a). At this stage, the embryo sac is already cellularized and consists of seven cells: three antipodal cells situated at the chalazal haustorium, which degenerate rapidly (frequently only their residues in the form of three highly stained spots could be observed, however, also non-degenerated antipodal cells could be observed); the central cell formed by two polar nuclei was observed just below the antipodals and they were beside each other, being very similar in shape and size (Figure 4b) (also see Additional file 2: Figures S2 and S4). Finally, the egg apparatus composed of one egg cell and two synergids was observed at the micropylar end (Figure 4a, b and c).

### Egg apparatus

**Figure 5:**
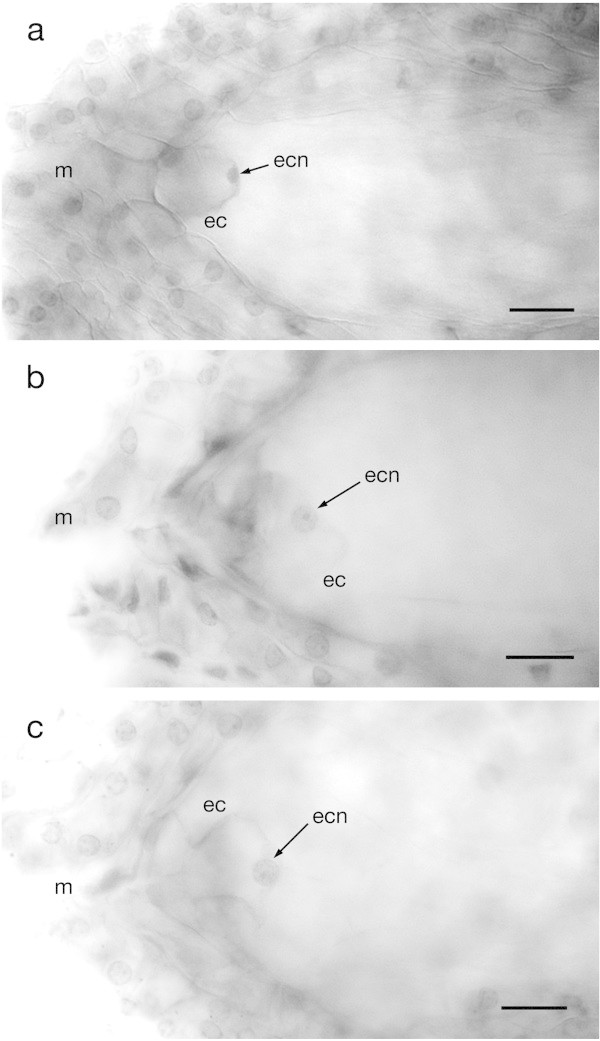
**Different egg cell shapes of** ***Agave tequilana*** **Weber. a)** Typical ovoid-shaped egg cell showing a highly condensed nucleus located at the chalazal end of the embryo sac. **b)** and **c)** Egg cells of an irregular shape with nucleus located at the chalazal end of the embryo sac. m = micropyle, ec = egg cell, ecn = egg cell nucleus. Bars = 20 μm.

Both synergids were found located at the micropylar end next to each other and generally observed in the same focal plane, while the egg cell was usually in a different focal plane and positioned between the two synergids. These three cells composing the ovular apparatus of *Agave tequilana* had their walls in contact with the micropylar edge of the female gametophyte (Figure 4d). The synergids are very similar to each other, and each cell possesses a vacuole polarized towards the chalazal end and the nucleus polarized towards the micropylar end (see Aditional file 2: Figure S3). The egg cells showed diverse morphologies probably due to different developmental stages at the moment of observation, however, in general, they had a highly dense nucleus located towards the chalazal extreme and a large vacuole occupying almost the whole space of the cell located towards the micropylar end of the sac with a size of about 28 μm long and 23 μm wide (Figure 5).

### The central cell: karyogamy of the polar nuclei

At the second day after pollination, the analyzed ovules did not show any change in size or morphology; however, the fusion of the two polar nuclei (karyogamy) to form the nucleus of the central cell was observed. This karyogamy in *Agave tequilana* occurs before the process of double fertilization (Figure 4c). The central cell nucleus remained located at the same position where both polar nuclei were observed at the chalazal extreme of the embryo sac. The central cell nucleus showed semi-circular to ovate morphologies with an approximate size of 17 μm long and 13 μm wide, and the approximate distance between the nucleus of the egg cell and that of the central cell was 210 μm.

### Endosperm and zygote formation

**Figure 6:**
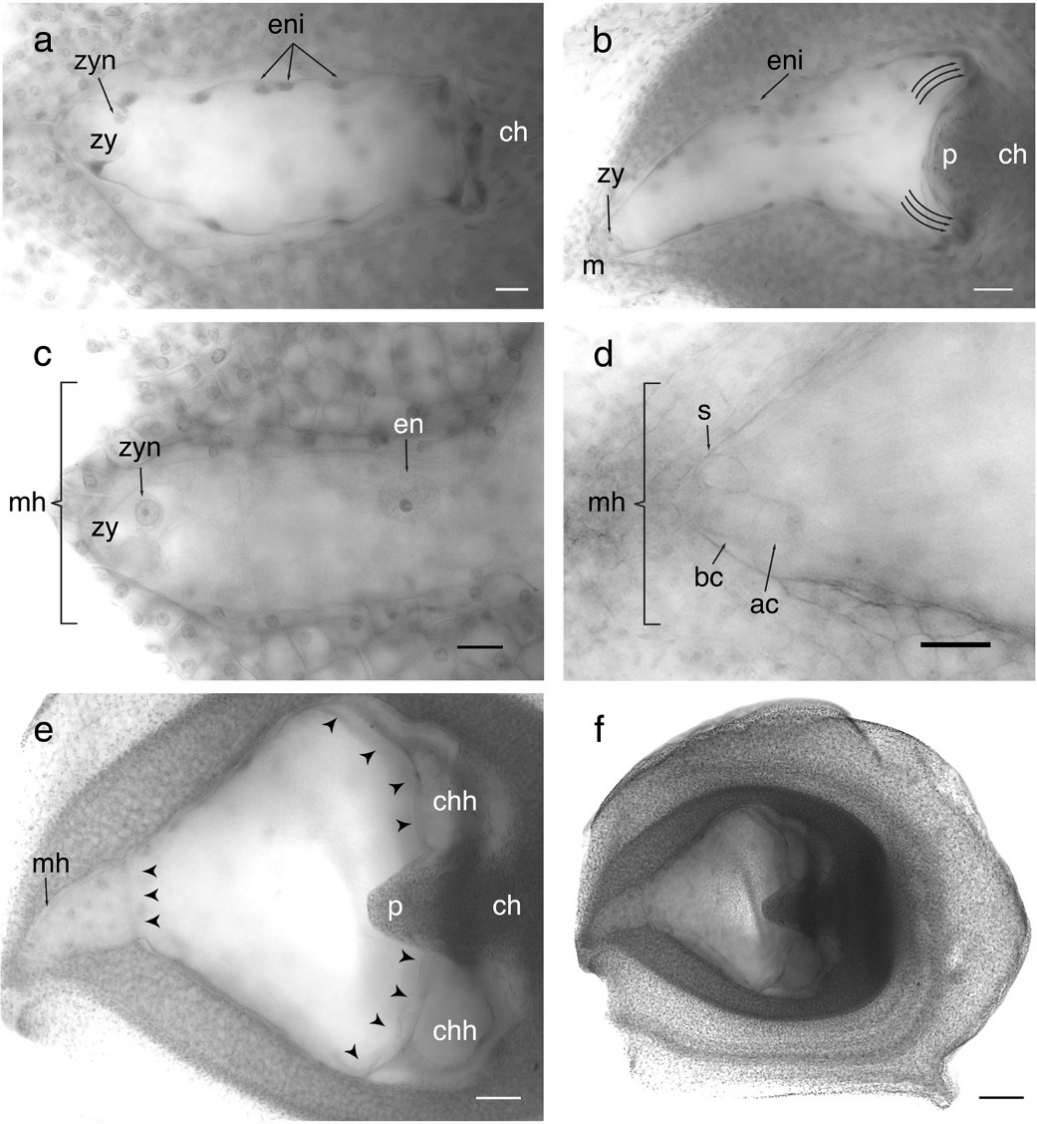
**Early embryo development of** ***Agave tequilana*** **Weber. a)** Zygote and early endosperm development. Bar = 10 μm. **b)** Formation of the chalazal haustorium (curved arrows) Bar = 50 μm. **c)** Formation of the micropylar haustorium. Bar = 25 μm. **d)** First division of the zygote showing the apical and basal cells of the early embryo. Bar = 25 μm. **e)** and **f)** Immature seed showing the micropylar haustorium and the chalazal haustorium including the postament. Bar in e = 100 μm and Bar in f = 200 μm. ch = chalaza, m = micropyle, zy = zygote, zyn = zygote nucleus, eni = endosperm nuclei, p = postament, mh = micropylar haustorium, chh = chalazal haustorium, en = endosperm nucleus, s = synergid, bc = basal cell, ac = apical cell. Curved arrows = haustoria pushing towards the chalaza, arrowheads = endosperm chambers. (for more detail, see close-up in Additional file 2: Figure S5).

After the double fertilization took place at three DAP (to be published elsewhere), the embryo sac started to increase its size and changed its morphology, thus at five DAP, the embryo sac had increased its size to about 280 μm long and 125 μm wide. At the same time, the embryo sac walls near the chalazal end and those that surround the haustorial tube begin to move towards the nucellar tissue (Figure 6a).

The zygote formation could be observed as the result of the fertilization of the egg cell by one of two sperm nuclei. The zygote possessed a hemispherical shape of 40 μm long and 38 μm wide, and a well polarized nucleus towards the chalazal pole of the embryo sac (Figure 6a). At the same time of zygote formation, the formation of disperse cells of the endosperm could be observed (Figure 6a,b, c). The endosperm of *Agave tequilana* was of the helobial type. The first mitotic division of the primary endosperm nucleus forms a cell wall, which generates two cells: a small cell at the chalazal extreme and a large cell at the micropylar extreme that occupies most of the embryo sac (see Additional file 2: Figure S4).

### Zygotic development and post-fertilization changes of the embryo sac

At six DAP, the *Agave tequilana* zygote enlarges almost 50% maintaining its original width. Meanwhile, the endosperm nuclei continued dividing and were mainly located at the central and chalazal regions of the embryo sac. Several nuclear divisions of the endosperm could be observed before the first division of the zygote occurred. Likewise, the embryo sac changes in form and size radically, its shape is irregular and resembles that of a heart, the walls surrounding the haustorial chalazal tube still pushing towards the chalaza thus forming two haustoria: a new one on each side of the chalazal end of the sac divided by a remnant of tissue called “postament” which in turn included the hypostase which was connected to the former chalazal haustorium (Figure 6b). Similarly, at the micropylar extreme starts the formation of the micropylar haustorium where the zygotic embryo is developed (Figure 6c,d,e). At this stage of development, it was possible to observe well defined chambers, one at the micropylar end and two at the chalazal end (Figure 6e).At eight and nine DAP the first division of the zygote could be observed. This first division gives rise to two cells: one basal cell and one apical cell that would form the embryo proper. Similarly to the zygote nucleus, the embryo apical cell showed a high polarity directed to the chalazal extreme. This polarity that is preserved from the egg cell, resulted in the asymmetric division of the zygote, thus the apical cell was smaller than the basal cell, and possessed a big and well-defined nucleus, while the basal cell seemed to be highly vacuolated (Figure 6d). At this point, the fertilized embryo sac is an immature seed ready toward maturity (Figure 6f).

## Discussion

### Megasporogenesis

The chalaza-micropyle linear orientation of tetrads in other species of this family has been previously reported in *Yucca rupicola* (Watkins 1937) and *Agave lechuguilla* (Grove 1941). However, Wolf (1940) reported the “T” formation of tetrads as the most frequent arrangement in *Y. aloifolia*, while Regen (1941) and Cave (1948) reported that the formation of meiotic tetrads may take either the “T” or the linear shape in *A. virginica* and *Hesperocallis undulata* respectively.

In *Agave tequilana*, only the closest megaspore cell to the chalazal pole remains viable (Monosporic-type) becoming the functional megaspore (FM). This is the case of most angiosperms including *Arabidopsis thaliana* and *Zea mays* (Yang et al. 2010) and the close related species *Agave virginica* (Regen 1941) and *Yucca aloifolia* (Wolf 1940). However, this megaspore is not always the one that survives, Reed (1903) reported that is the second closest megaspore the one that remains viable and is converted to a FM in *Y. filamentosa*. On the other hand, Piven et al. (2001) observed that the embryo sac of *Agave fourcroydes* and *A. angustifolia* develops from the two megaspores closest to the micropylar pole originating an embryo sac of the bisporic Allium-type.

### Megagametogenesis

The size of the functional megaspore (43 μm long and 25 μm wide) found in the present study was similar to the dimensions reported for *Tofieldia glutinosa* where the size of the FM was 35–50 μm long and 12–20 μm wide (Holloway and Friedman 2008). The hypostase located at the chalazal pole of the embryo sac in the nucellar tissue is reported as frequent among members of the Asparagaceae (formerly Agavaceae) family (Tilton and Mogensen 1980), and it is probable that it plays an important function in the translocation of nutrients from the ovule to the gametophyte before and after fertilization (Tilton 1980). Furthermore, the three synchronized mitotic divisions without cytokinesis of the functional megaspore were similar to many reported in the literature i.e. in the formation of the maize embryo sac (Huang and Sheridan 1994).

### The mature embryo sac

The haustorial tube at the chalazal end of the embryo sac observed in *Agave tequilana* resembles to that reported by Tilton (1978) for *Ornithogalum*. This haustorial tube may play a role for some kind of a nutritious function and penetrates the nucellar tissue close to the hypostase and the vascular strands, which come from the funiculus (Reed 1903; Watkins 1937; Wolf 1940; Rudall 1997).

The cellularized embryo sac consisted of seven cells (eight nuclei): three antipodal cells situated at the chalazal end (only their residues could be observed); two polar nuclei close to the antipodals and the egg apparatus containing one egg cell and two synergids. Occasionally, sacs with less than eight nuclei are found in many species and this can be due to a rapid degeneration of the antipodals or they may go unnoticed because they are hidden at the end of the chalazal tube (Maheshwari 1948, 1950). This phenomenon of rapid disintegration of the antipodal cells has been reported for several plant species, such as *Agave virginica* (Regen 1941), *Glycine max* (Kennell and Horner 1985), *Scilla persica* (Svoma and Greilhuber 1988), *Triticum aestivum* (Zhang et al. 1988; An and You 2004), *Arabidopsis thaliana* (Murguia et al. 1993), *Passiflora edulis* (Magalhães de Souza et al. 2002), *Sargentodoxa cuneata* (Wang et al. 2009) and *Cichorium intybus* (Chehregani et al. 2011).

According to Tilton (1978), in angiosperms, antipodals are cells that vary in their behavior in the mature megagametophyte and the only trait they share with each other is their location in the chalazal end of the sac; the antipodals can be ephemeral, degraded shortly after their formation or persist even after fertilization (Williams and Friedman 2004). In *Tofieldia glutinosa*, antipodals can even proliferate in the maturation stage of the embryo sac, being up to eight antipodal nuclei (Holloway and Friedman 2008). Polar nuclei in *Agave tequilana* were observed close to the antipodals remains and being similar in shape and size as reported by Maheshwari (1950) and Tilton and Lersten (1981). The position of the polar nuclei is similar to that observed in *Hemiphylacus alatostylus* (Rudall et al. 1997) which are located in the chalazal end within a tube or neck (chalazal haustorium), and in *Tofieldia glutinosa* where polar nuclei are located at a two-thirds distance from the micropylar end of the embryo sac (Holloway and Friedman 2008).

Moreover, the close contact of the egg apparatus with the micropyle is highly similar to what was observed in the ovular apparatus of *Ornithogalum caudatum* (Tilton 1978), and the polarization of the nucleus of both synergids towards the micropylar end was similar to what was reported for tobacco (Tian et al. 2005). Furthermore, as in many other angiosperms, the egg cell possesses a highly dense nucleus located towards the chalazal extreme of the embryo sac (Tilton 1978; Mogensen and Suthar 1979; Tian et al. 2005).

### The central cell: karyogamy of the polar nuclei

It was observed that in *Agave tequilana*, karyogamy of the polar nuclei to form the diploid nucleus of the central cell occurred before the process of double fertilization, similar to that observed in ovules of *Capsella bursa-pastoris* (Schulz 1973) and *Tofieldia glutinosa* (Holloway and Friedman 2008). This diploid nucleus remained at the same place where both polar nuclei were observed at the chalazal extreme of the embryo sac. This chalazal position of the central cell nucleus has been observed in *Yucca rupicola* (Watkins 1937), *Persea americana* (Tomer and Gottreich 1976) and *Tofieldia glutinosa* (Holloway and Friedman 2008) among others. However, Piven et al. (2001) reported for *Agave fourcroydes* and *A. angustifolia* that the position of the polar nuclei and finally the nucleus of the central cell were located in the center of the embryo sac; or close to the egg apparatus at the micropylar end, a pattern that is present in almost all angiosperms (Tilton 1978; Russell 1993), particularly in *Zea mays* (Huang and Sheridan 1994) and in *Arabidopsis thaliana* (Olsen 2004).

### Endosperm and zygote formation

Double fertilization gives rise to both, the zygote and the endosperm. In this work the typical polarization of the zygote nucleus towards the chalazal pole could be observed. This polarization of the zygote nucleus resembled those of *Capsella bursa-pastoris* (Schulz and Jensen 1968), *Nicotania tabacum* (Mogensen and Suthar 1979) and *Arabidopsis thaliana* (Mansfield and Briarty 1991; Mansfield et al. 1991).

In this study, the helobial type of endosperm development was similar to the one reported for *Hesperocallis undulata* (Cave 1948) which has the central cell nucleus at the chalazal extreme of the embryo sac just below of the antipodal cell remains located at the chalazal tube. Maheshwari (1950) reported that when the position of the central cell nucleus is located close to the antipodals, the endosperm type of development will be helobial.

### Zygotic development and changes post-fertilization in the embryo sac

At six DAP, the *Agave tequilana* zygote enlarged almost 50% maintaining its original shape, this enlargement has been previously reported for *Arabidopsis thaliana* (Bowman et al. 1994) among many others, and the endosperm cells continued dividing before the first division of the zygote. These observations are similar to those in *Amaranthus hypochondriacus* (Coimbra and Salema 1999), suggesting that the central cell is precocious in regard to its development after fertilization. Around these days, the embryo sac suffers drastic changes in size and with the formation of the micropylar haustorium where the zygote is located and with the formation of two chalazal haustoria. There exist several reports about the formation of chalazal haustoria in species belonging to the Asparagales, where the endosperm development invades lateral sections of the proximal nucellus, destroying the lateral tissue and leaving the “postament” at the center of the sac (Rudall 1997).

Finally, at nine DAP the first division of the zygote takes place giving rise to one basal cell and one apical cell which is the first cell of the embryo proper. This observed process was similar to what is described for the majority of angiosperms (Lau et al. 2012). Furthermore, the polarity of the embryo apical cell toward the chalazal extreme of the embryo sac occurred similarly as in most of flowering plants (Rodríguez-Garay et al. 2000).

## Conclusions

The *Agave tequilana* embryo sac development is a monosporic Polygonum-type, showing ephemeral antipodals. In the present work, the detailed embryo sac development, the formation of the zygote, the early embryo formation and the helobial type of endosperm are reported for the first time in this species. However, further ultrastructural studies are needed for a more detailed knowledge. The results reported here show basic knowledge about the early embryo development and allow new paths for basic and applied research for systematic and evolutionary studies and breeding programmes where *in vitro* fertilization, selfing, and intra- and inter-generic hybridization are needed.

## Methods

*Agave tequilana* presents perfect flowers with six tepals and anthers, an inferior ovary that is divided in three locules, each locule containing two rows of numerous anatropous ovules with axillary placentation (Gentry 1982). The plant material used in this study consisted of inflorescences collected from mature plants growing in the state of Jalisco, which is located in the Tequila appellation of origin in Mexico (DOF 1977), from May to June in the years 2010–2013.

At the beginning of the flowering season, panicles that contained flower buds with different development stages were collected (five panicles per inflorescence or plant). Afterwards, ovules from these young buds were extracted from the ovary using fine forceps and knives under a dissection microscope, and then fixed in a FAA solution (10:5:50:35 formaldehyde: acetic acid: ethanol; distilled water) for 24 hours. After fixation, ovules were transferred to a 70% ethanol solution and stored at 5°C for later staining.

In order to maintain cross-pollination to ensure fertilization and embryo formation, the rest of the flower buds were emasculated before anthesis and removed anthers were kept at room temperature until they matured after one or two days. Mature pollen was recovered and stored in a desiccator at 4°C for future pollinations. Once stigmas were receptive, 10 mature non-pollinated flowers were selected per each panicle and their ovules were extracted from the ovaries, they were processed following the procedure used for buds. The rest of the flowers with receptive stigmas were hand pollinated using a small paintbrush. Cross-pollination was carried out since auto-pollination did not assure fertilization. In order to study the embryo sac and zygotic embryo development, 10 immature fruits were collected from panicles at 2, 3, 4, 5, 6, 8 and 9 days after pollination (DAP).

Ovules and immature seeds, previously fixed in FAA and kept in 70% ethanol, were stained based on the technique reported by Stelly et al. (1984), in short, a Mayer’s-Hematoxylin solution was used for staining during a 10–24-hour period and then treated with a 2% acetic acid solution during a 16-hour period in order to eliminate the excess stain. Ovule samples were washed with a 0.1% sodium bicarbonate solution until the solution was clear. At this point, ovules were left in the same 0.1% sodium bicarbonate solution for 24 hours at room temperature before dehydration. Afterwards, samples were dehydrated in an ethanol series of 25%, 50%, 70%, 85%, 95% and 100% during 15 minutes, and finally, in 100% ethanol during 2 hours. Clarification was carried out through a series of methyl salicylate:ethanol solutions of 3:1, 1:1, 1:3, for one hour each.

Clarified ovules were mounted in a methyl salicylate solution for microscope observation. The samples were analyzed using a Leica DMR microscope (Wetzlar, Germany) coupled to an Evolution QEi camera (Media-Cybernetics, Bethesda, USA). Images were taken using Image Pro® software (Media-Cybernetics, Bethesda, USA), and microphotographs were processed with the Adobe Photoshop Software version CS6 and evenly adjusted for better contrast.

## Electronic supplementary material

Additional file 1: Table S1: Mean size of the different developmental stages in the analized ovules of *Agave tequilana*. (PDF 158 KB)

Additional file 2: Figure S1: Structure of the ovule of *Agave tequilana*. **Figure S2.** The antipodal cells and the central cell nucleus of *Agave tequilana*. **Figure S3.** The synergid cells of *Agave tequilana*. **Figure S4.** Helobial endosperm development in *Agave tequilana*. **Figure S5.** Close-up of a two celled embryo of *Agave tequilana* showing a large vacuolated basal cell. (PDF 1 MB)
