# Supplementary material for: Embryo sac formation and early embryo development in Agave tequilana (Asparagaceae)
Source: Springerplus. 2014 Oct 1;3:575. doi: 10.1186/2193-1801-3-575 (PMC4192144; doi:10.1186/2193-1801-3-575)
Supplement: Supplementary file 1 — Additional file 1: Table S1: Mean size of the different developmental stages in the analized ovules of Agave tequilana. (PDF 158 KB) [file 40064_2014_1272_MOESM1_ESM.pdf]

Table S1. Mean size of the different developmental stages in the analized ovules of *Agave tequilana.*

Developmental stages	Number of observed ovules	Mean size±S.D. (μm).	
		Long	Wide
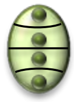 **Tetrad**	32	58.06±2.913	21.38±0.894
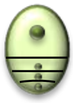 **Functional megaspore**	21	33.41±1.305	23.47±0.539
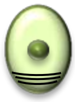 **Functional megaspore**	30	43.27±2.218	25.03±0.784
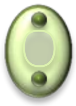 **Two nuclei embryo sac**	40	50.46±3.578	35.15±1.834
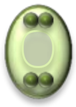 **Four nuclei embryo sac**	48	60.00±3.411	42.04±1.359
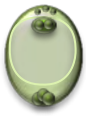 **Mature embryo sac**	182	247.04±6.873	106.12±7.592
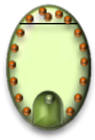 **Embryo sac at 5DAP**	20	280.24±10.054	125.08±2.362

Table S1. Continued.

Cells in the mature and fertilizated embryo sac	Number of observed ovules	Mean size±S.D. (µm).	
		Long	Wide
Central cell nucleus	162	17.01 ±0.593	13.06±0.836
Egg cell	82	28.25±2.223	23.10±3.114
Distance between the egg cell and the endosperm cell nucleus in the mature embryo sac	82	210.03±16.707	
Zygote at 5DAP	20	40.24±6.141	38.36±7.797
