# Supplementary material for: Embryo sac formation and early embryo development in Agave tequilana (Asparagaceae)
Source: Springerplus. 2014 Oct 1;3:575. doi: 10.1186/2193-1801-3-575 (PMC4192144; doi:10.1186/2193-1801-3-575)
Supplement: Supplementary file 2 — Additional file 2: Figure S1: Structure of the ovule of Agave tequilana. Figure S2. The antipodal cells and the central cell nucleus of Agave tequilana. Figure S3. The synergid cells of Agave tequilana. Figure S4. Helobial endosperm development in Agave tequilana. Figure S5. Close-up of a two celled embryo of Agave tequilana showing a large vacuolated basal cell. (PDF 1 MB) [file 40064_2014_1272_MOESM2_ESM.pdf]

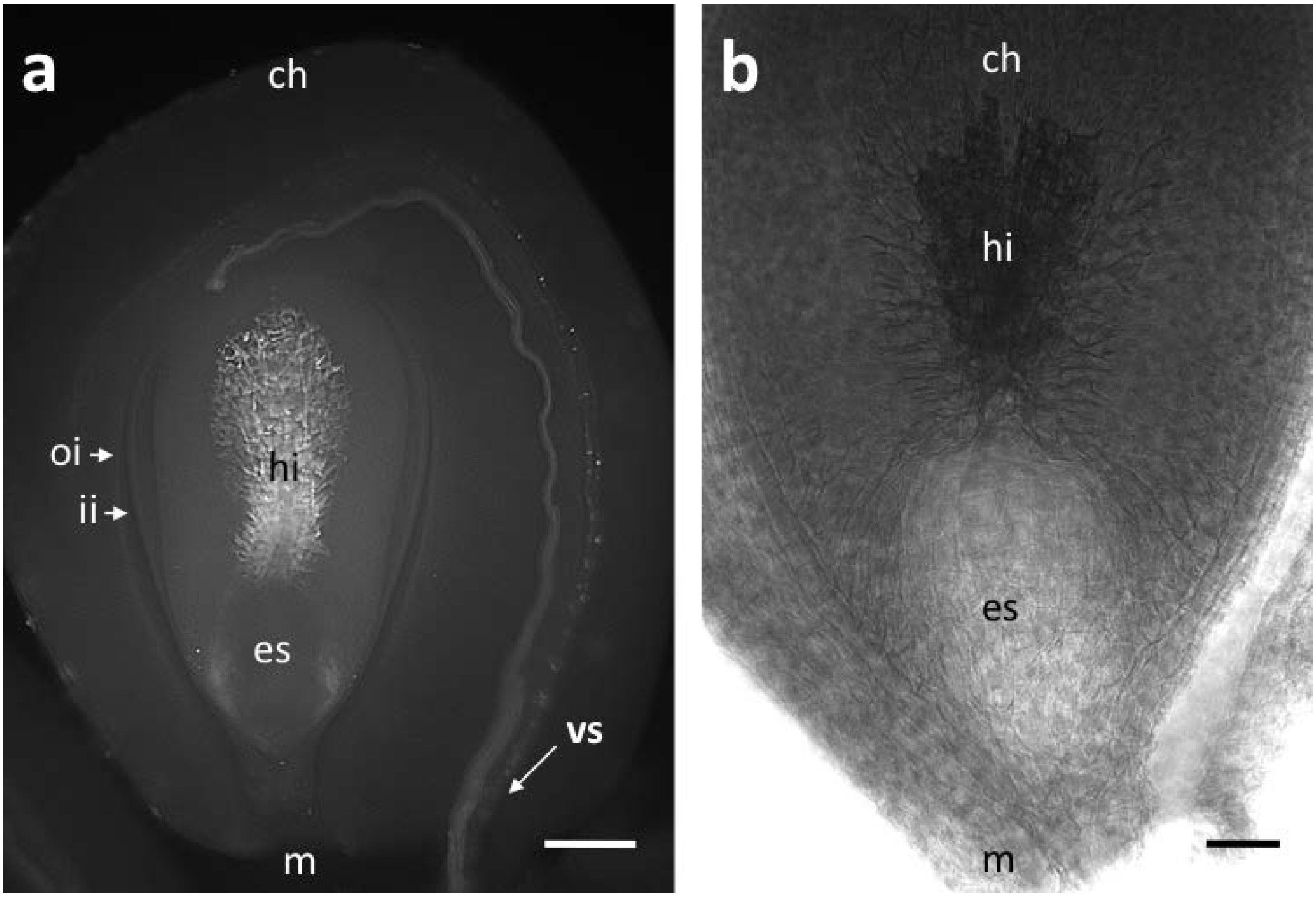

Figure S1. Structure of the ovule of *Agave tequilana*. a) Immature ovule stained with aniline blue. The hypostase is highly fluorescent under epifluorescense microscopy. Bar=100µm. b) Immature ovule stained as described in the Methods section of this paper. The hypostase is highly stained with hematoxilin. Bar=40µm. ch=chalaza, m=micropyle, oi=outer integument, ii=inner integument, hi=hypostase, es=embryo sac, vs=vascular strand.

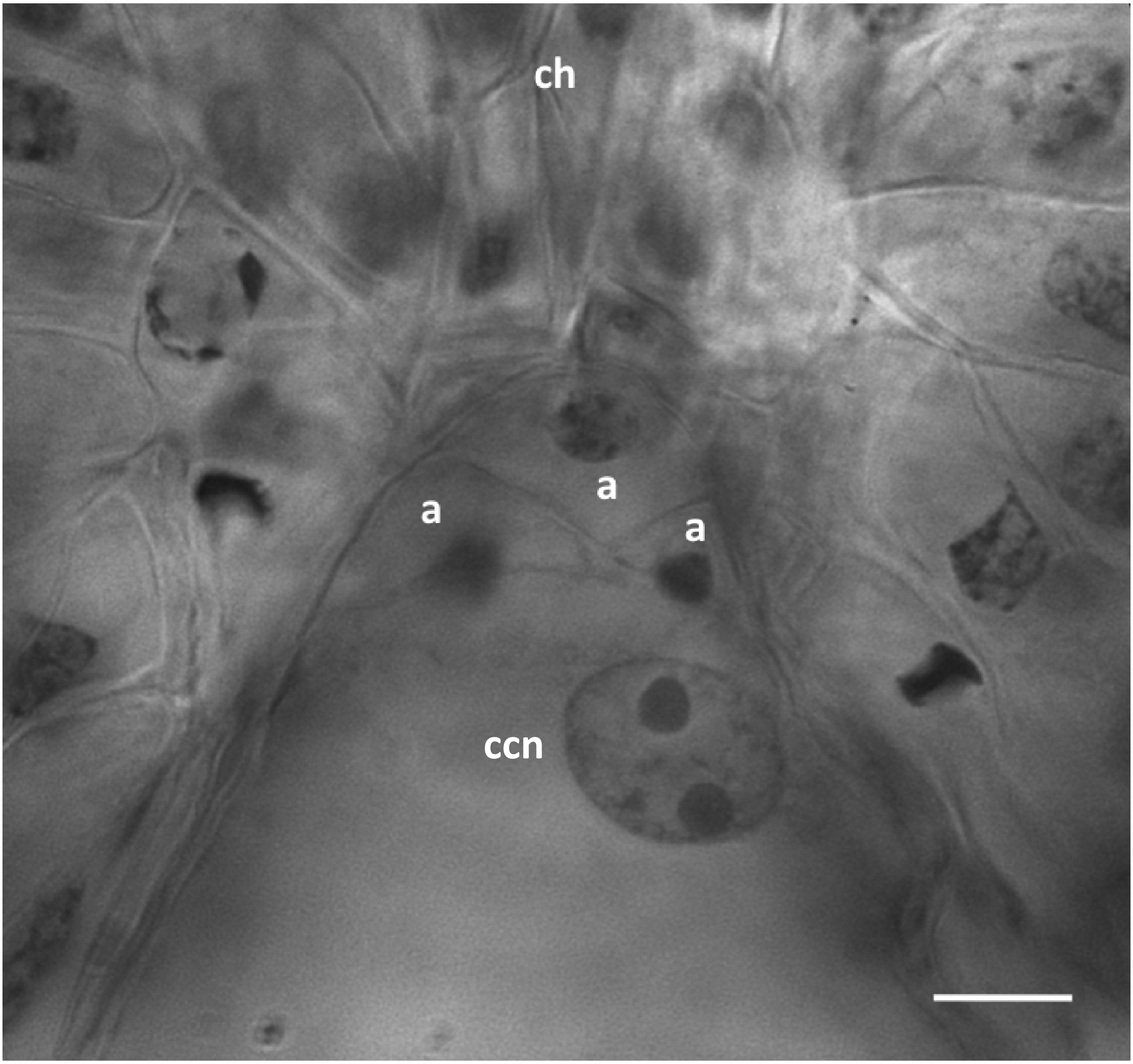

Figure S2. The antipodal cells and the central cell nucleous of *Agave tequilana*. a=non-degenerated antipodal cells with intact cell wall. ccn=central cell nucleus before karyogamy of polar nuclei.

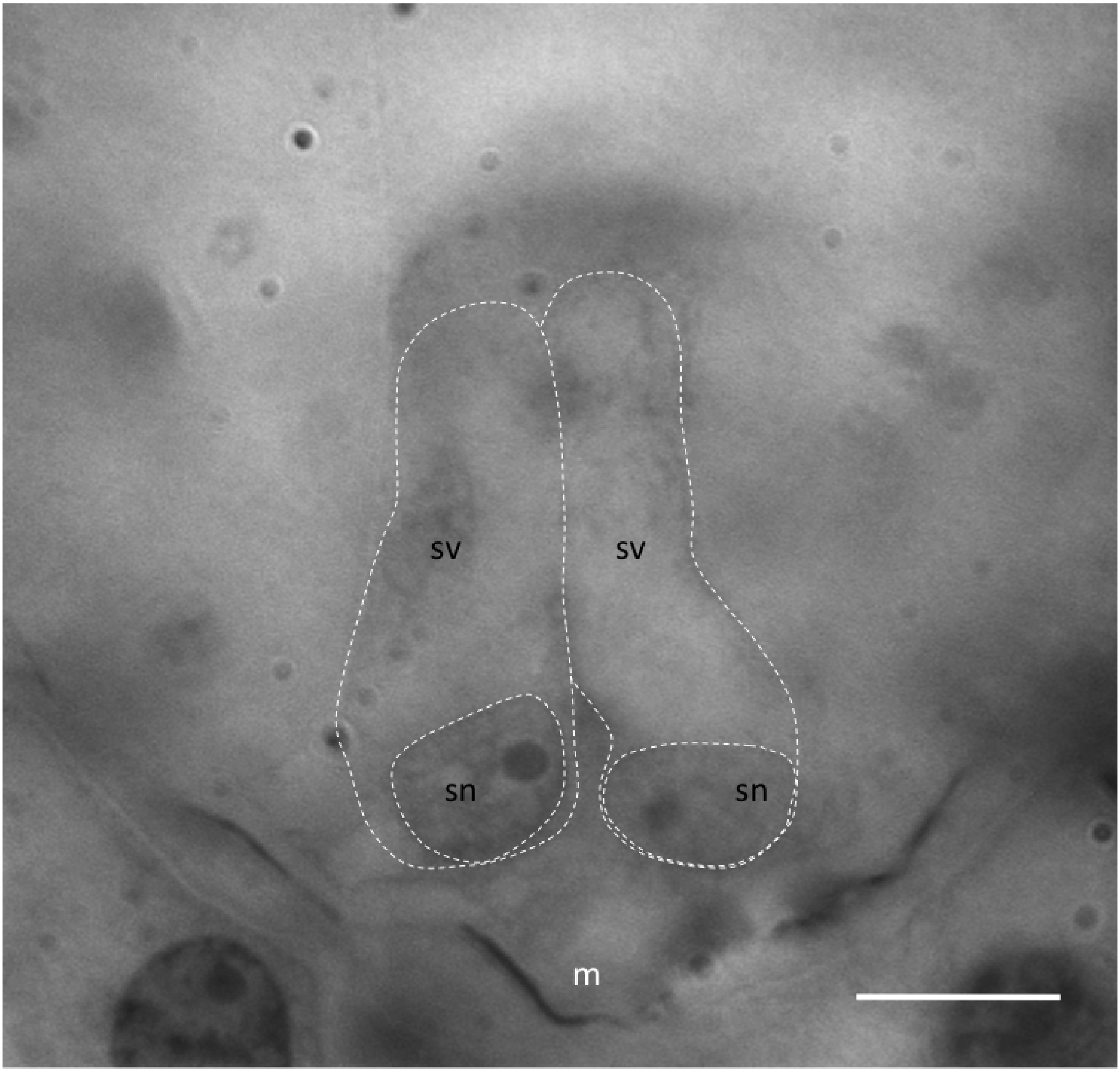

Figure S3. The synergid cells of *Agave tequilana*. Nuclei are positioned at the micropylar pole and vacuoles are positioned at the chalazal pole of the embryo sac.

syv=synergid vacuole, syn=synergid nucleus, m=micropylar pole. Bar=10µm.

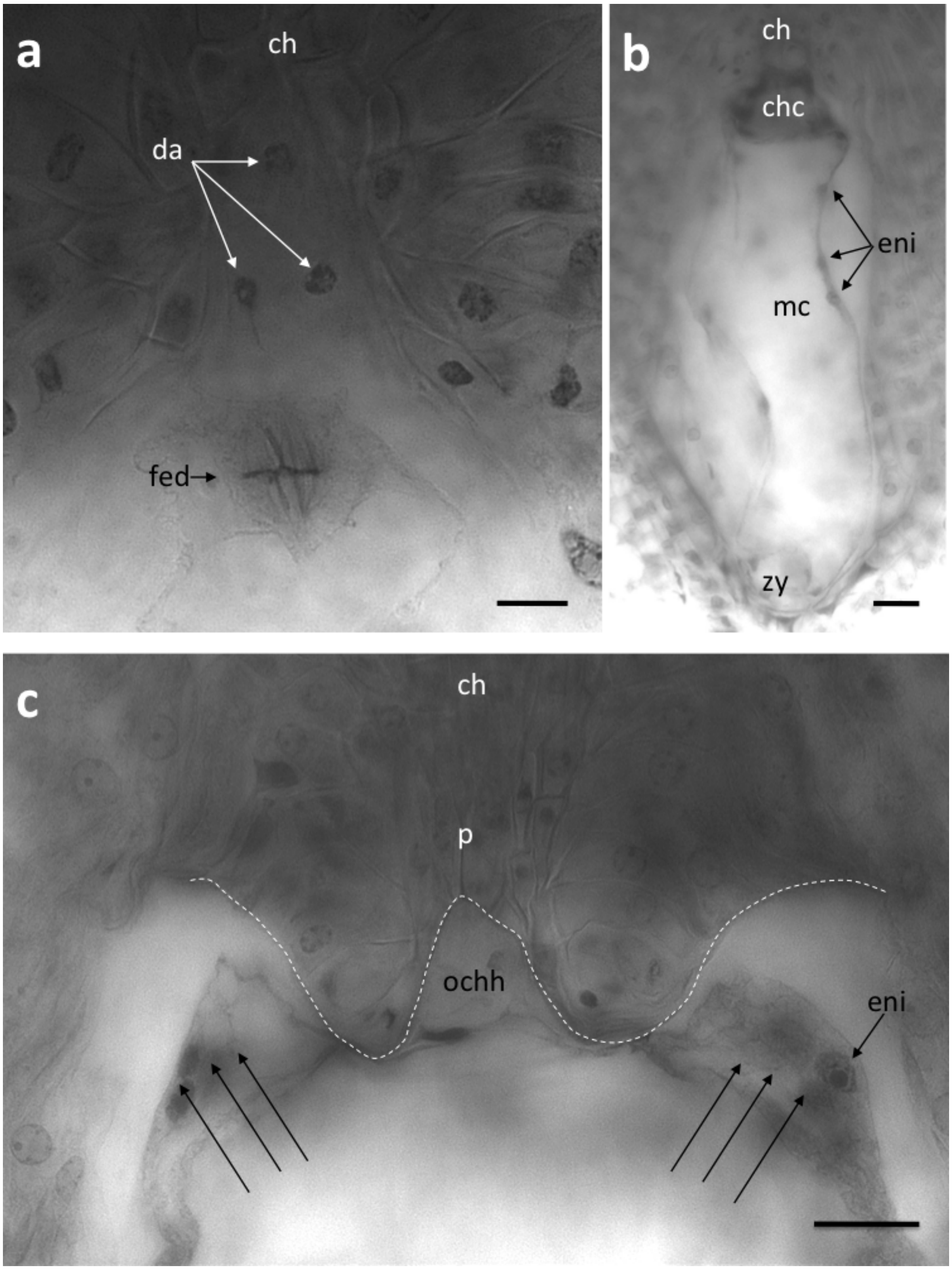

Figure S4. Helobial endosperm development in *Agave tequilana.* a) First mitotic division of the primary endosperm nucleous at the chalazal pole of the embryo sac. Bar=10µm. b) Formation of a small chalazal chamber and a large micropylar chamber resulting from the first mitotic division. Bar=20µm. c) Advanced stage of endosperm development. Bar=20µm. fed=first endosperm mitotic division, da=degenerating antipodals, ch=chalaza, eni=endosperm nuclei, ochh=original chalazal hustorium, p=postament, arrows=haustoria pushing towards the chalaza.

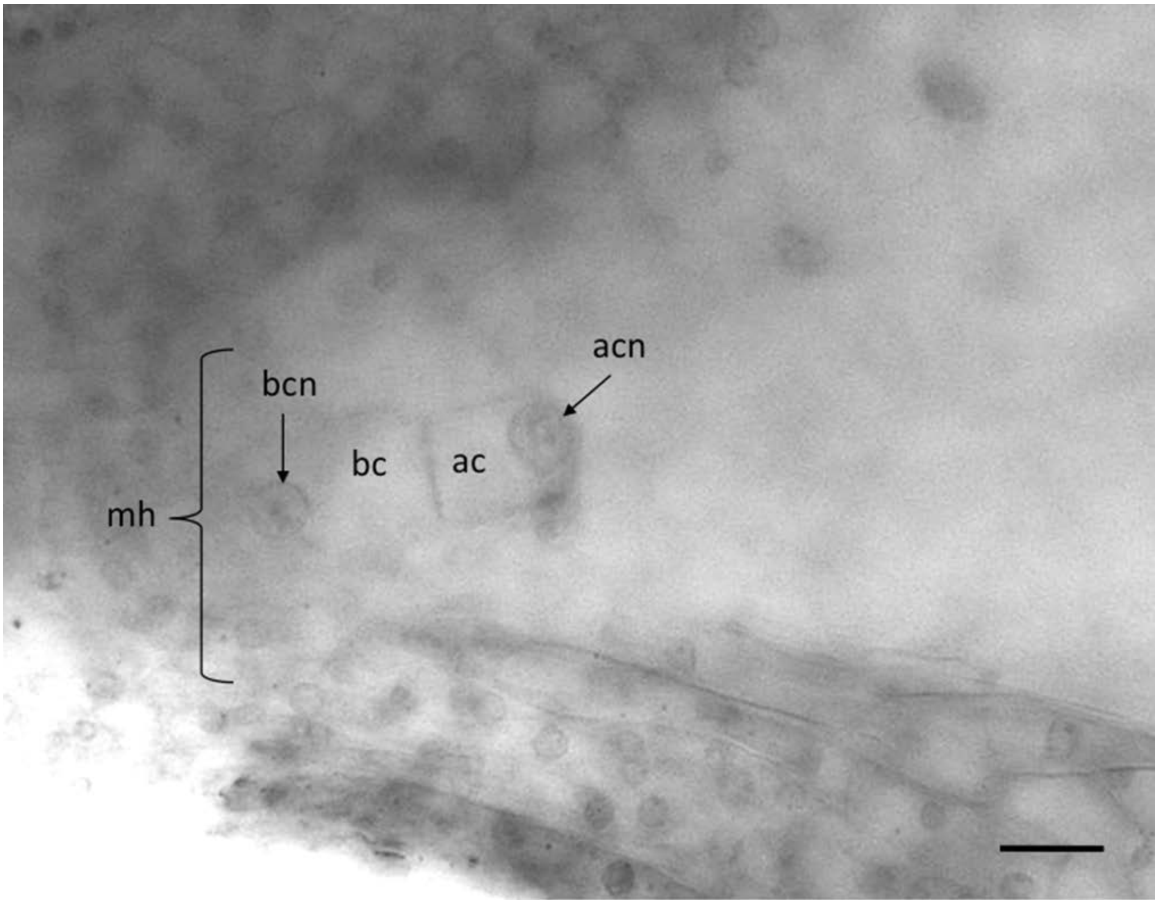

Figure S5. Close-up of a two celled embryo of *Agave tequilana* showing a large vacuolated basal cell. (as in Figure 6d of the paper). mh=micropylar haustorium, bcn=basal cell nucleus, ac=apical cell, acn=apical cell nucleus. Bar=20µm.
